# Gut microbial bile acid metabolite skews macrophage polarization and contributes to high-fat diet-induced colonic inflammation

**DOI:** 10.1080/19490976.2020.1819155

**Published:** 2020-10-02

**Authors:** Lingyu Wang, Zizhen Gong, Xiuyuan Zhang, Fangxinxing Zhu, Yuchen Liu, Chaozhi Jin, Xixi Du, Congfeng Xu, Yingwei Chen, Wei Cai, Chunyan Tian, Jin Wu

**Affiliations:** a Department of Pediatric Surgery, Xinhua Hospital, School of Medicine, Shanghai Jiaotong University, Shanghai, China; b Department of Gastroenterology and Nutrition, Shanghai Institute for Pediatric Research, School of Medicine, Shanghai Jiaotong University, Shanghai, China; c Shanghai Key Laboratory of Pediatric Gastroenterology and Nutrition, Shanghai, China; d State Key Laboratory of Proteomics, Beijing Proteome Research Center, National Center for Protein Sciences(Beijing), Beijing Institute of Lifeomics, Beijing, China; e Department of Cardiology, Shanghai Jiao Tong University Affiliated Sixth People’s Hospital, Shanghai, China; f Department of Immunology, Shanghai Institute of Immunology, Shanghai Jiaotong University School of Medicine, Shanghai, China

**Keywords:** High fat diet, microbiota, colonic inflammaion, bile acid, macrophage polarization, toll-like receptor

## Abstract

High-fat diet (HFD) leads to systemic low-grade inflammation, which has been involved in the pathogenesis of diverse metabolic and inflammatory diseases. Colon is thought to be the first organ suffering from inflammation under HFD conditions due to the pro-inflammatory macrophages infiltration, however, the mechanisms concerning the induction of pro-inflammatory phenotype of colonic macrophages remains unclear. In this study, we show that HFD increased the percentage of gram-positive bacteria, especially genus *Clostridium*, and resulted in the significant increment of fecal deoxycholic acid (DCA), a gut microbial metabolite produced by bacteria mainly restricted to genus *Clostridium*. Notably, reducing gram-positive bacteria with vancomycin diminished fecal DCA and profoundly alleviated pro-inflammatory macrophage infiltration in colon, whereas DCA-supplemented feedings to vancomycin-treated mice provoked obvious pro-inflammatory macrophage infiltration and colonic inflammation. Meanwhile, intra-peritoneal administration of DCA also elicited considerable recruitment of macrophages with pro-inflammatory phenotype. Mechanistically, DCA dose-dependently promoted M1 macrophage polarization and pro-inflammatory cytokines production at least partially through toll-like receptor 2 (TLR2) transactivated by M2 muscarinic acetylcholine receptor (M2-mAchR)/Src pathway. In addition, M2-mAchR mediated increase of TLR2 transcription was mainly achieved via targeting AP-1 transcription factor. Moreover, NF-κB/ERK/JNK signalings downstream of TLR2 are involved in the DCA-induced macrophage polarization. In conclusion, our findings revealed that high level DCA induced by HFD may serve as an initiator to activate macrophages and drive colonic inflammation, thus offer a mechanistic basis that modulation of gut microbiota or intervening specific bile acid receptor signaling could be potential therapeutic approaches for HFD-related inflammatory diseases.

## Introduction

High-fat diet (HFD) consumption could induce systemic chronic low-grade inflammation, termed meta-inflammation, which has been linked to diverse metabolic and inflammatory abnormalities, such as type II diabetes, atherosclerosis and inflammatory bowel disease (IBD).^1–3^ Long-term high-fat diet feeding could break intestinal immune homeostasis and induce inflammation in animal model, and epidemiological studies have also shown that excessive high-fat diet intake is closely related to the occurrence and relapse of IBD.^1,4–8^ More importantly, recent studies indicate that colon may be the first organ suffering from inflammation caused by high-fat diet, which attributes to the colonic infiltration of pro-inflammatory macrophages.^9^

As an important integral compartment of the intestinal immune system, macrophages play critical roles in pathogen clearance, inflammation regulation and local homeostasis maintaining. According to the different activation modes, macrophages exhibit either a pro-inflammatory M1 phenotype (classical activated), or an anti-inflammatory M2 phenotype (alternative activated). M1 macrophages could secret pro-inflammatory cytokines such as TNF-α, IL-6, IL-1β and produce nitric oxide (NO), participating in inflammatory response and eliminating pathogens, thus execute immune surveillance functions; while M2 macrophages generally produce anti-inflammatory factors, such as IL-10 and TGF β, which promote tissue repair and remodeling.^10–12^ Imbalance of M1/M2 macrophage polarization contributes to the occurrence and development of IBD.^13–17^ Previous studies have proved that macrophages massively accumulated in the inflamed colon tissue and M1 phenotype was predominant in IBD patients.^18^ Moreover, Kawano and colleagues reported that short-term high-fat diet could increase the number of pro-inflammatory macrophages in the lamina propria of colon, accompanied by increased expression levels of pro-inflammatory cytokines such as TNF-α and IL-1β, which highlighted the critical role of M1 macrophages in the HFD-related colonic inflammation.^9^ However, what drives the pro-inflammatory (M1) phenotype of colonic macrophages under the HFD settings remains largely unclear.

The colon holds trillions of microbes and it has been suggested that microbial metabolites contribute to the orchestration of immune response and gut homeostasis.^19–21^Accumulating evidence indicates that HFD induces the alteration of gut microbiota, which could lead to the increased production of secondary bile acids, especially deoxycholic acid (DCA), an important gut bacterial metabolite reported to participate in the occurrence of IBD and gastrointestinal cancer dose-dependently.^22–24^ As the major secondary bile acid, DCA is produced exclusively through 7α-dehydroxylation reactions by bacteria mainly belonging to Genus *Clostridium* (such as *Clostridium* cluster XI and XIVa, Gram-positive bacteria) in the human colon and undergoes reabsorption to portal vein system by passive diffusion.^25^ Fecal DCA concentration can increase nearly tenfold in individuals on a high fat diet, and furthermore, long-term feeding of animal with a DCA-supplemented diet could induce obvious colonic inflammation similar to human IBD.^7,26^ Considering that reabsorbed DCA accumulates in the colonic lamina propria during back to portal circulation, we reasoned that under the HFD settings colonic macrophages could encounter high level of DCA which may provoke the pro-inflammatory phenotype switch of macrophages. Therefore, we sought to explore a possible link between the HFD-induced increase of microbial metabolite DCA and macrophage polarization as well as colonic inflammation.

In the present study, we provide evidence that excessive DCA correlates with HFD-induced colonic pro-inflammatory macrophages infiltration and tissue injury. DCA can dose-dependently promote M1 macrophage polarization and pro-inflammatory cytokines production at least in part through TLR2 signaling transactivated by M2-mAchR/Src pathway. M2-mAchR mediated up-regulation of TLR2 transcription by DCA is mainly achieved via targeting AP-1 transcription factor.

## Results

### Vancomycin treatment alleviates HFD-induced colonic pro-inflammatory macrophages infiltration

**Figure 1.**
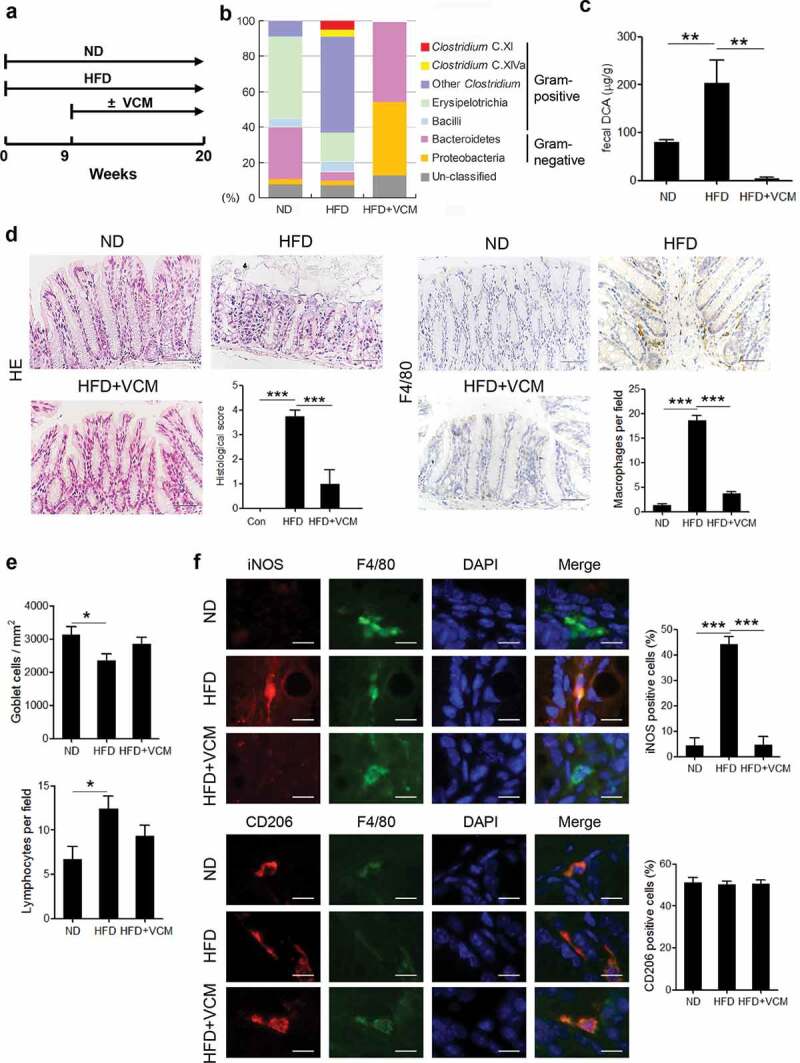
Vancomycin treatment changes gut microbiota and alleviates HFD-induced colonic pro-inflammatory macrophages infiltration. (a) Animal treatment procedure (ND, n = 5; HFD, n = 6; HFD+VCM, n = 6). (b) Stool samples were analyzed by 16S rDNA sequencing and the relative abundance of OTUs in the fecal bacterial community is shown. Data represent the mean of three mice per group. (c) Fecal DCA concentrations. (d) Representative HE and F4/80 staining as well as histological score of colon sections of differently treated mice (Scale bar, 50 µM). Quantification of IHC staining was exhibited as the number of F4/80 positive cells per field (n = 5). (e) Quantification of the number of goblet cells per mm^2^ (upper panel) and lymphocytes per field (bottom panel). (f) Representative iNOS (red), F4/80 (green), CD206 (red) and DAPI (blue) immunofluorescence staining of colon tissues from ND, HFD and HFD plus vancomycin treated mice (Scale bar, 10 µM). The histograms indicate the percentages of iNOS or CD206 positive cells in macrophages (F4/80^+^). **: *p* < .01; ***: *p* < .001. Error bars indicate s.e.m.

To ascertain the role of microbiota alteration in the HFD-associated colonic inflammation, wild – type C57BL/6 mice were fed with HFD for 20 weeks and some of which were treated with vancomycin after the 9th week (Figure 1(a)). Compared to the normal diet, intestinal microbiome analysis revealed that HFD feedings could dramatically increase the percentage of Gram-positive bacteria, especially *Clostridium* species including *Clostridium* clusters XI and XVIa (Figure 1(b)), meanwhile, the amount of fecal DCA was also significantly elevated (Figure 1(c)). Moreover, HFD induced obvious macrophage infiltration and tissue damage in the colon, and notably, the colonic macrophages in HFD-fed mice expressed more iNOS but not CD206 compared with the counterparts in the normal diet-fed mice. (Figure 1(d–f)). Importantly, vancomycin (VCM) treatment which preferentially targets Gram-positive bacteria efficiently decreased the fecal DCA, accompanied by profound inhibition of M1 macrophages infiltration and intestinal damage (Figure 1(b–f)). These data suggest that the increase of Gram-positive bacteria-associated elements may be involved in the colonic pro-inflammatory macrophages infiltration under HFD conditions.

### Bacterial metabolite DCA contributes to HFD-induced colonic pro-inflammatory macrophages infiltration and inflammatory injury

**Figure 2.**
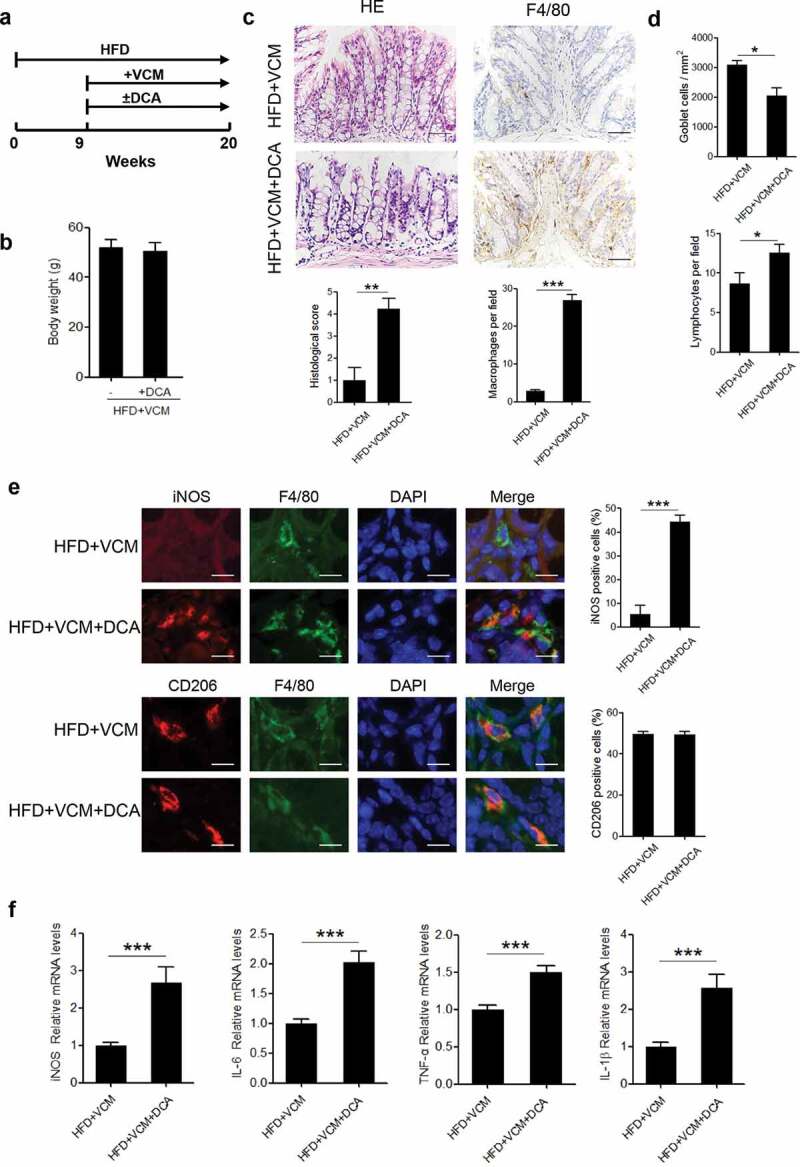
DCA contributes to HFD-induced colonic pro-inflammatory macrophages infiltration and inflammatory injury. (a) Animal treatment procedure (n = 6 per group). (b) Body weight. (c) Representative HE and F4/80 staining as well as histological score of colon sections from HFD+VCM and HFD+VCM+DCA treated mice (Scale bar, 50 µM). The numbers of F4/80 positive cells per field were quantified (n = 5). (d) Quantification of the number of goblet cells per mm^2^ (upper panel) and lymphocytes per field (bottom panel). (e) iNOS (red), F4/80 (green), CD206 (red) and DAPI (blue) immunofluorescence analysis of colon sections from HFD+VCM and HFD+VCM+DCA treated mice (Scale bar, 10 µM). Percentages of iNOS or CD206 positive cells in macrophages (F4/80^+^) were shown. (f) Real-time PCR analysis of iNOS, IL-6, TNF-α and IL-1β levels in colonic homogenates (n = 4 per group). **: *p* < .01; ***: *p* < .001. Error bars indicate s.e.m.

Considering the alteration of fecal DCA level paralleled with the changes of colonic inflammatory injury, we then attempted to explore whether DCA increment could trigger pro-inflammatory phenotype of colonic macrophages. To this end, DCA feeding was performed in HFD-fed mice treated with vancomycin (Figure 2(a,b)). Intriguingly, DCA feeding alone in VCM-treated HFD-fed mice was observed to induce substantial pro-inflammatory macrophages (iNOS^+^) infiltration and increase the expression of multiple pro-inflammatory factors (IL-6, TNF-α, IL-1β, iNOS) in the colon tissue (Figure 2(c–f)), mimicking the inflammatory intestinal damage induced by HFD. These results indicate that the excessive DCA produced under HFD settings may be involved in the HFD-induced colonic inflammation.

Since accumulating studies have demonstrated that HFD increases sensitivity to chemical – induced or genetic colitis models, we further observed whether HFD-related high level DCA contributes to the increased susceptibility of mice to DSS-induced colonic injury. In line with the previous reports, HFD-fed mice treated with DSS underwent more severe colonic tissue damage than those on a normal-chow diet, as evidenced by much shortening of colon length, higher histopathological score and increased production of pro-inflammatory factors in colon. Vancomycin (VCM) administration effectively ameliorated above-mentioned intestinal damage in HFD-fed DSS-treated mice, whereas DCA-supplemented feed reversed the therapeutic effect of VCM and resembled the tissue inflammation in HFD-fed DSS-treated counterparts (Supplementary Figure 1). These findings suggest that high level colonic DCA caused by HFD may participate in the increased predisposition to intestinal inflammation.

### DCA induces M1 macrophage polarization

**Figure 3.**
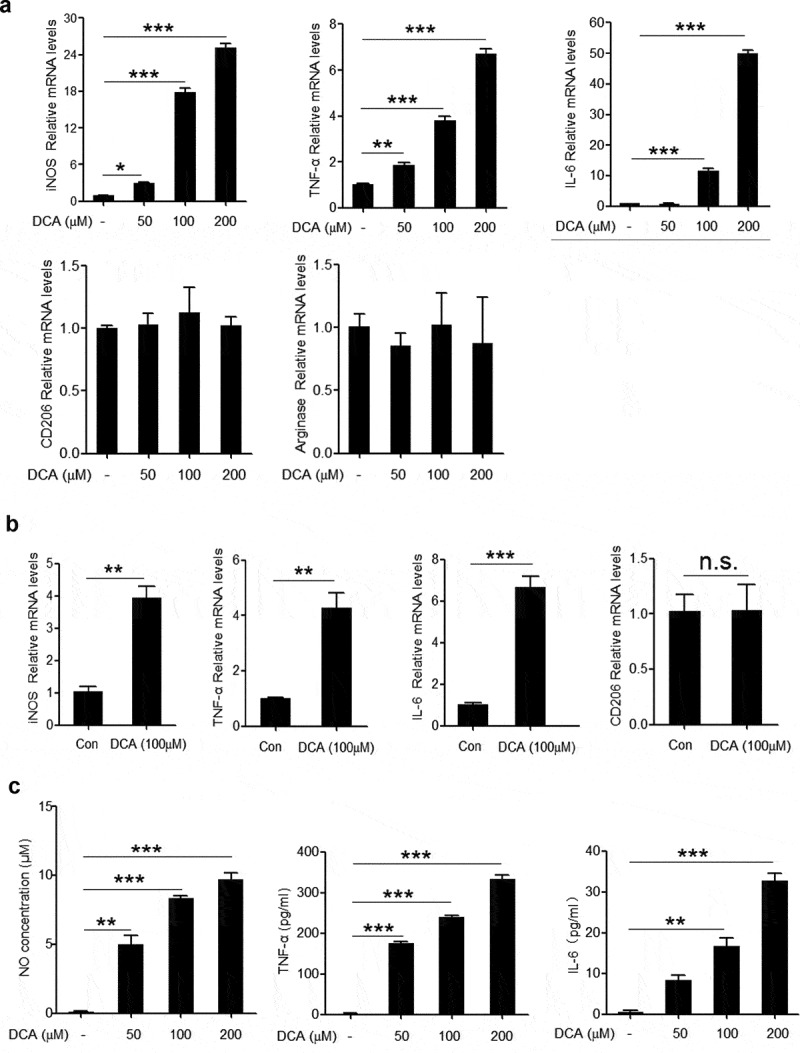
DCA induces M1 macrophage polarization *in vitro*. (a) RAW264.7 macrophages were treated with DCA (0, 50, 100, 200 µM) for 4h. mRNA expression levels of iNOS, TNF-α, IL-6, CD206 and arginase were analyzed by real-time PCR. (b) Bone marrow derived macrophages (BMDMs) were treated with or without DCA (100 µM) for 4 h. mRNA expression levels of iNOS, TNF-α, IL-6 and CD206 were determined by real-time PCR. (c) RAW264.7 macrophages were treated with DCA (0, 50, 100, 200 µM) for 24 h. NO levels in the supernatant were detected by Griess reagents. TNF-α and IL-6 in culture medium were analyzed by ELISA.*: *p* < .05; **: *p* < .01; ***: *p* < .001. n.s.: no statistically significant difference (*p* > .05). Representative data from 3 independent experiments giving similar results are shown. Error bars indicate s.e.m.

Given the critical role of M1 macrophages in the HFD-induced colonic inflammation, we then verified whether DCA was able to modulate macrophage polarization *in vivo*. To this end, C57BL/6 mice were intra-peritoneally injected with DCA or vehicle and peritoneal cells were collected for the analysis of macrophage recruitment and phenotype transition. The results showed that DCA administration elicited a considerable increase in the recruitment of macrophages (Supplementary Figure 2a), meanwhile, macrophages from DCA-injected mice produced much higher NO and TNF-α compared with controls (Supplementary Figure 2b). In addition, the phenotype of peritoneal macrophages was further confirmed by flow cytometry analysis showing that more iNOS expression, but not CD206, was observed in macrophages from DCA-injected mice compared to the controls (Supplementary Figure 2c). To further confirm the effect of DCA on macrophage polarization *in vitro*, murine macrophage cell line RAW264.7 was stimulated with different dosage of DCA, and we found that the mRNA expression levels of M1 macrophage signature genes including iNOS, TNF-α and IL-6, were obviously up-regulated in a dose-dependent manner in response to DCA (Figure 3(a)). In contrast, the expression of M2 macrophage-associated genes (CD206 and arginase) was not significantly affected upon DCA stimulation (Figure 3(a)). Meanwhile, DCA exhibited the similar effects on murine bone marrow derived macrophages (BMDMs) (Figure 3(b)). In line with the mRNA levels, NO, TNF-α and IL-6 concentrations in the cell culture supernatant were also dramatically increased after DCA treatment (Figure 3(c)). Moreover, DCA had no significant impact on macrophage viability under our experiment conditions (Supplementary Figure 3a). Collectively, these data suggest that DCA could promote macrophage polarization toward a pro-inflammatory M1 phenotype.

### DCA-induced macrophage polarization is mediated by M2 muscarinic acetylcholine receptor (M2-mAchR)

**Figure 4.**
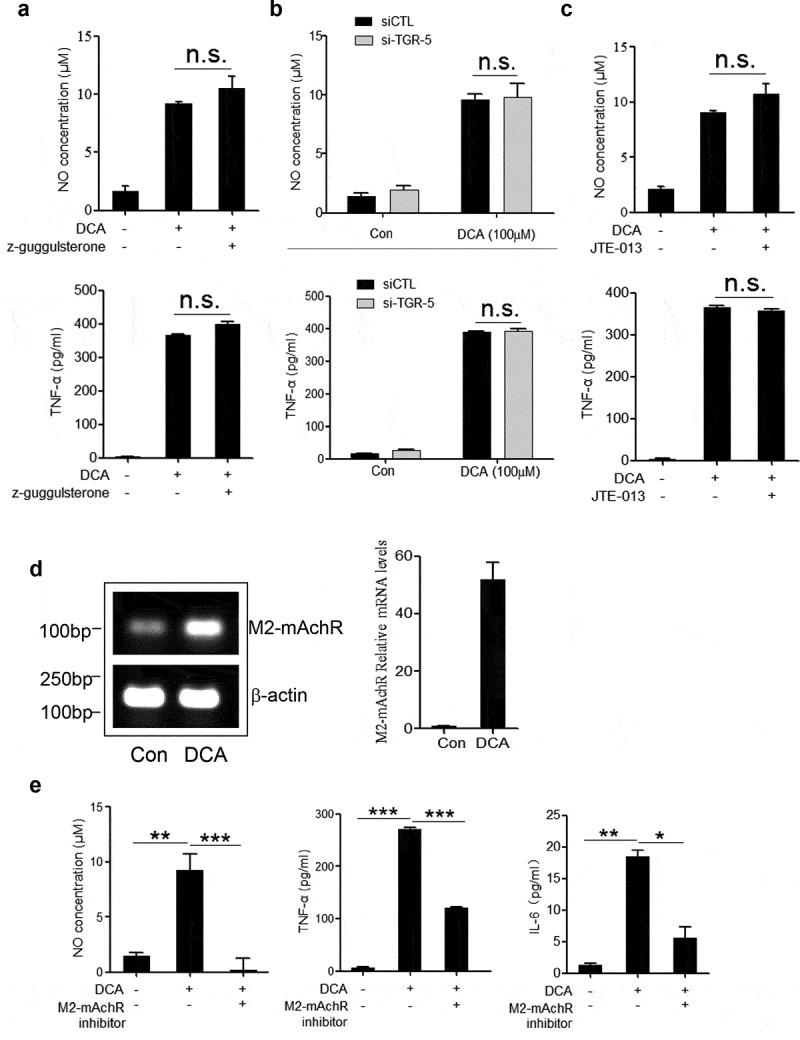
DCA-induced macrophage polarization is mediated by M2 muscarinic acetylcholine receptor (M2-mAchR). (a) RAW264.7 macrophages were stimulated with DCA (100 µM) in the presence or absence of Z-Guggulsterone (20 μM). NO and TNF-α in supernatants were analyzed. (b) Scramble control siRNA (siCTL) or TGR5 siRNA (siTGR5) transfected RAW264.7 macrophages were stimulated with DCA (100 µM). NO and TNF-α in supernatants were analyzed. (c) RAW264.7 macrophages were stimulated with DCA (100 µM) in the presence or absence of JTE-013 (10 μM). NO and TNF-α in supernatants were determined. (d) RAW264.7 macrophages were stimulated with DCA (100 µM) for 4 h. The expression of M2-mAchR was measured by regular PCR and real-time PCR. (e) RAW264.7 macrophages were stimulated with DCA (100 µM) in the presence or absence of M2-mAchR inhibitor (methoctramine, 5 µM). NO, TNF-α and IL-6 in supernatants were analyzed. *: *p* < .05; **: *p* < .01; ***: *p* < .001. n.s.: no statistically significant difference (*p* > .05). Representative data from 3 independent experiments are shown. Error bars indicate s.e.m.

To elucidate the molecular mechanism underlying the DCA-induced macrophage polarization, bile acid receptors including farnesoid X-receptor (FXR), TGR5 and Sphingosine-1-phosphate receptor 2 (S1PR2) were inhibited respectively.^27,28^ The results showed that suppression of any bile acid receptors mentioned above had no obvious effect on TNF-α and NO production in response to DCA (Figure 4(a–c) and Supplementary Figure 4a-b), suggesting that other kind of receptors may participate in the induction of M1 macrophage polarization. Unexpectedly, M2 muscarinic acetylcholine receptor (M2-mAchR), which has emerged as a receptor of conjugated bile acid,^28^ appeared to play an important role. Indeed, DCA stimulation could induce potent increase of mRNA expression level of M2-mAchR in macrophages, and selective inhibition of M2-mAchR significantly reduced the production of TNF-α, NO and IL-6 triggered by DCA (Figure 4(d,e) and Supplementary Figure 4c). These data indicate that DCA-induced macrophage polarization is largely mediated by M2-mAchR.

### Toll-like receptor 2 (TLR2) is up-regulated upon DCA stimulation and involved in DCA-induced macrophage polarization

**Figure 5.**
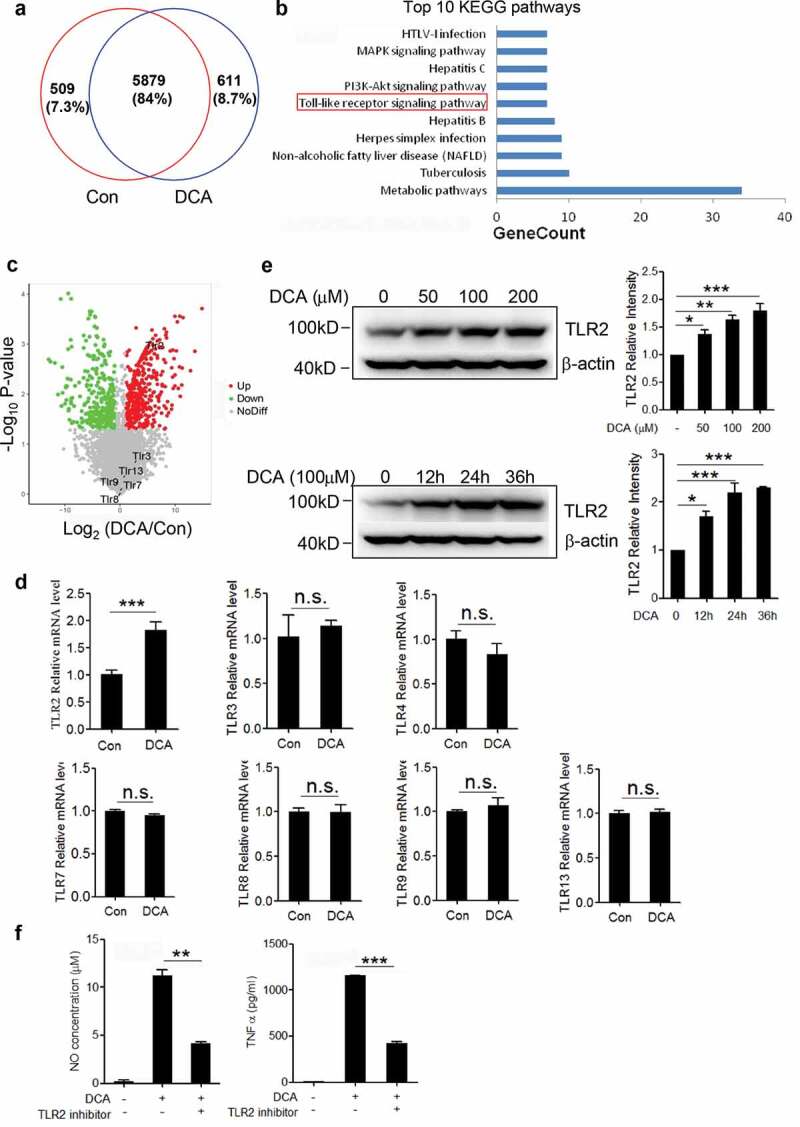
Toll-like receptor 2 (TLR2) is up-regulated upon DCA stimulation and involved in DCA-induced macrophage polarization. (a) Venn diagram of the whole cell proteins from untreated and DCA-treated BMDMs. (b) Top 10 enriched KEGG pathways for up-regulated proteins by DCA treatment. (c) Volcano plot of differentially expressed proteins (DEPs). Red and green dots represent up-regulated and down-regulated DEPs upon DCA treatment, respectively. Grey dots indicate the unchange proteins. (d) RAW264.7 macrophages were treated with or without DCA (100 µM) for 4 h. The expression of TLRs was determined by real-time PCR. (e) RAW264.7 macrophages were treated with DCA (0, 50, 100, 200 µM) for 24 h or treated with 100 µM DCA for indicated time points. The protein expression levels of TLR2 were measured by immunoblot and β-actin was regarded as a loading control. The relative intensity of the bands was quantitated. (f) RAW264.7 macrophages were stimulated with DCA (100 µM) in the presence or absence of TLR2 inhibitor (C29, 100 µM) for 24 h. NO and TNF-α in supernatants were analyzed. *: *p* < .05; **: *p* < .01; ***: *p* < .001. n.s.: no statistically significant difference (*p* > .05). Data are representative of three independent experiments with similar results. Error bars indicate s.e.m.

To further explore the possible molecules involved in the DCA-induced macrophage polarization, murine bone marrow derived macrophages (BMDMs) were stimulated with or without DCA and differential protein expression was analyzed (Figure 5(a–c)). Importantly, KEGG signaling pathways analysis for all proteins that were up-regulated upon DCA treatment revealed that ‘Toll-like receptor signaling pathway’, which plays a key role in innate immunity and inflammatory response, is significantly enriched (Figure 5(b)). In the volcano plot, Toll-like receptor 2 (TLR2) was identified as the most potently altered TLRs being 6.7-fold increased by DCA (Figure 5(c)). To confirm the relationship between TLRs expression and DCA treatment, real-time PCR was implemented on control and DCA-treated macrophages. Consistent with the proteomic data, the mRNA expression level of TLR2 but not other TLRs was substantially up-regulated upon DCA stimulation (Figure 5(d)). Western-blot analysis also verified that DCA could increase TLR2 protein expression in a dose – and time-dependent manner (Figure 5(e)). More importantly, TLR2 specific inhibitor could strongly suppress DCA-induced TNF-α and NO production in macrophages (Figure 5(f) and Supplementary Figure 4d). Therefore, our proteomic and functional analysis lead us to propose that TLR2 signaling should be critical in the process of DCA-induced M1 macrophage polarization.

### DCA enhances TLR2 transcription by targeting AP-1 via M2-mAchR

**Figure 6.**
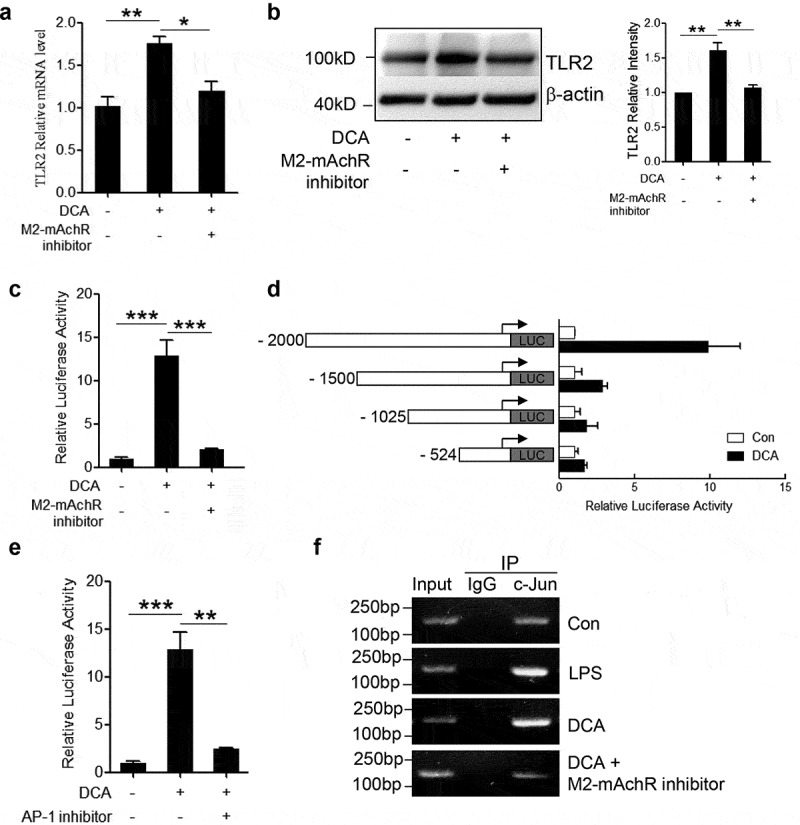
DCA regulates TLR2 transcription by targeting AP-1. RAW264.7 macrophages were stimulated with DCA (100 µM) in the presence or absence of methoctramine (5 µM). TLR2 level was determined by (a) real-time PCR or (b) western blot. The relative intensity of the TLR2 bands was quantitated. (c) RAW264.7 macrophages were transfected with a TLR2 promoter reporter construct, then left untreated or treated with DCA (100 μM) in the presence or absence of methoctramine (5 µM). Luciferase activities were measured and normalized by Renilla activity. (d) RAW264.7 macrophages were transfected with a series of deletion mutants of TLR2 promoter reporter plasmids, followed by DCA or vehicle treatment. Luciferase activities were measured and normalized. (e) RAW264.7 macrophages were transfected with a TLR2 promoter reporter construct, then left untreated or treated with DCA (100 μM) in the presence or absence of AP-1 inhibitor (SR 11302, 100 µM). Luciferase activities were determined. (f) RAW264.7 macrophages were stimulated with DCA (100 µM) in the presence or absence of methoctramine (5 µM), followed by ChIP assay. LPS (1 µg/ml) was used as a positive control. Anti-c-Jun antibody or isotype-matched IgG control antibody were used. PCR was applied to quantify the precipitated DNA with primers flanking the AP-1 binding region of the TLR2 promoter. *: *p* < .05; **: *p* < .01; ***: *p* < .001. Error bars indicate s.e.m. Representative data from 3 independent experiments are shown.

To figure out the connection between bile acid receptor M2-mAchR and TLR2 expression under DCA stimulation, murine macrophages were treated with DCA in the presence or absence of M2-mAchR selective inhibitor, mRNA and protein levels of TLR2 were determined by real-time PCR and western blot respectively. Indeed, M2-mAchR blockage almost reduced TLR2 expression to the baseline level (Figure 6(a,b)). To better understand the regulatory mechanism of TLR2 expression directed by DCA, luciferase activity assay was conducted by using a 2000-bp TLR2 promoter reporter plasmid. As expected, luciferase activity markedly increased upon DCA stimulation and was efficiently eliminated by M2-mAchR inhibitor (Figure 6(c)). The subsequent deletion analysis exhibited that deletion of −2000 to −1500 bp region significantly reduced the TLR2 promoter activity in response to DCA, suggesting that the critical DCA-related responsive element seems to be located in this distal portion of TLR2 promoter (Figure 6(d)). Notably, an important AP-1 binding site within above region was predicted by bioinformatics analysis, together with the observation showing that AP-1 inhibitor could greatly diminish the TLR2 promoter activity induced by DCA (Figure 6(e)), these data indicate that DCA regulates TLR2 transcription mainly by targeting AP-1. To confirm this result, chromatin immunoprecipitation (ChIP) assay was performed using a c-Jun-specific antibody. Data showed that c-Jun antibody pulled down much more AP-1 binding region sequences on TLR2 promoter in DCA-treated macrophages than untreated control, and consist with previous findings, M2-mAchR inhibitor substantially reversed this effect (Figure 6(f) and Supplementary Figure 5). In addition, both M2-mAchR inhibitor and AP-1 inhibitor used here have no obvious effect on macrophage viability (Supplementary Figure 3b).

### DCA promotes tyrosine phosphorylation of TLR2 and activates ERK/JNK/NF-κB signaling

**Figure 7.**
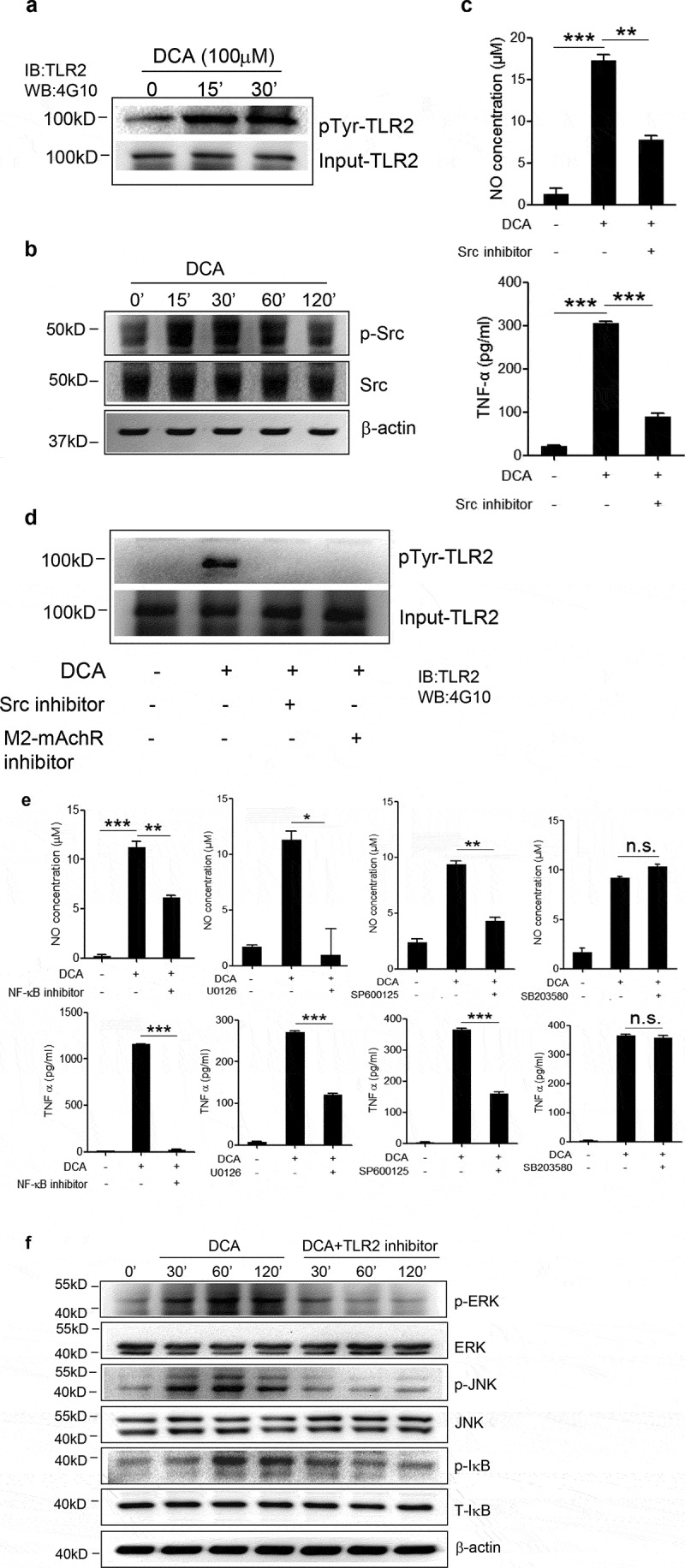
DCA promotes TLR2 tyrosine phosphorylation and activates its downstream signaling. (a) RAW264.7 macrophages were treated with DCA for indicated time points. Tyrosine phosphorylation of TLR2 was detected. (b) RAW264.7 macrophages were treated with DCA for indicated time points. phospho-Src (Tyr416), total Src and β-actin were detected by western blot. (c) RAW264.7 macrophages were stimulated with DCA (100 µM) in the presence or absence of SKI-606 (Src inhibitor, 10 nM). NO and TNF-α in supernatants were analyzed. (d) RAW264.7 macrophages were stimulated with DCA (100 µM) in the presence or absence of SKI-606 (10 nM) or methoctramine (5 µM) for 15 min. Tyrosine phosphorylation of TLR2 was detected. (e) RAW264.7 macrophages were stimulated with DCA (100 µM) in the presence or absence of QNZ (20 μM), U0126 (10 μM), SP600125 (25 μM) or SB203580 (10 μM) for 24 h. NO and TNF-α in supernatants were analyzed. (f) Immunoblot analysis of phospho-ERK, total ERK, phospho-JNK, total JNK, phospho-IκB and total IκB of RAW264.7 macrophages treated with DCA in the presence or absence of C29 (100 µM). β-actin was deemed as a loading control. *: *p* < .05; **: *p* < .01; ***: *p* < .001. n.s.: no statistically significant difference (*p* > .05). Data shown are representative of 3 individual experiments. Error bars indicate s.e.m.

We then sought to exam the effect of DCA stimulation on TLR2 signaling. The cytosolic domain of TLR2 contains several tyrosine residues, and tyrosine phosphorylation of TLR2 was reported to be required for NF-κB signaling activation by *Staphylococcus aureus*.^29^ Here we found that DCA could also induce obvious TLR2 phosphorylation at tyrosine residues (Figure 7(a)), implying the initiation of signaling cascades. Bile acids have been found to transactivate other receptors (eg. EGFR) via bile acid receptors, including mAchR.^30^ Additionally, Src, belonging to the nonreceptor Src family tyrosine kinases, was involved in the bacterial ligands and mechanical stress induced TLR2 tyrosine phosphorylation,^31,32^ meanwhile, it also played a critical role in the crosstalk between M2-mAchR and L-type Ca^2+^ channel,^33^ then we further investigated whether DCA transactivates TLR2 in a M2-mAchR-mediated manner, which involves Src. The results showed that DCA treatment significantly increased Src phosphorylation (Tyr416) (Figure 7(b)), and consistently, Src inhibition could strongly decrease DCA-induced TNF-α and NO production (Figure 7(c)). More importantly, M2-mAchR as well as Src blockage almost completely suppressed TLR2 tyrosine phosphorylation in response to DCA (Figure 7(d)), indicating that the activation of TLR2 by DCA was mainly mediated via M2-mAchR/Src signaling. Since MAPKs and NF-κB pathways downstream of TLR2 are considered to be critical in the regulation of inflammatory response, to verify their involvement in DCA-induced inflammation, each pathway was selectively inhibited. Data showed that blockage of NF-κB as well as ERK/JNK pathways could markedly abrogate TNF-α and NO production induced by DCA, while P38 inhibitor failed to abolish the pro-inflammatory role of DCA (Figure 7(e)). In line with these results, DCA treatment dramatically up-regulated the phosphorylation of ERK1/2, JNK and IκB, which were all greatly compromised by TLR2 inhibition (Figure 7(f)). These findings suggest that DCA-induced macrophage polarization involves TLR2-NF-κB/ERK/JNK pathway.

## Discussion

Systemic low-grade inflammation contributes to HFD-related disorders.^2^ Recent findings have shown that colonic inflammation precedes other tissues and ultimately results in the distal organ inflammation. Although evidence indicated that colonic inflammation could be induced by alteration of microbiota and mucosal immune homeostasis under HFD conditions, detailed mechanisms involved in the initiation of intestinal inflammation remains elusive. In this study, we proved that high colonic DCA level caused by the alteration of microbiota in mice on a HFD correlated with pro-inflammatory macrophages infiltration and inflammatory injury in colon. *In vitro* studies showed that DCA could dose-dependently promote M1 macrophage polarization and pro-inflammatory cytokines production. In addition, DCA-induced macrophage polarization is largely mediated by M2 muscarinic acetylcholine receptor (M2-mAchR), more intriguingly, TLR2 pathway is regulated by DCA-M2-mAchR axis and at least partially involved in the process of DCA-induced macrophage polarization. Collectively, these data offer a new mechanism that the alteration of microbial metabolite induced by HFD triggers colonic inflammation through promoting pro-inflammatory macrophage phenotype switch.

The gut microbiota has long been recognized as integral component for regulating intestinal homeostasis, while microorganisms and their metabolites are actively involved in diverse physiological and pathological processes in intestine. As the special microbial metabolites, the role of bile acids (BA) in modulating inflammatory and immune responses has recently emerged.^34^ Bile acids are synthesized in liver as primary BA (PBA), then stored in gallbladder and secreted into intestine.^35^ The majority of PBAs are reabsorbed in the distal ileum and back to the liver through portal vein system, and a small amount of PBAs (about 5%) escaping from ileum resorption are converted into secondary bile acids after entering the colon, which mainly include DCA and LCA, depending on the colonic bacteria. Moreover, DCA can be reabsorbed by passive diffusion through the colonic mucosa, whereas LCA is mostly excreted in stool.^36^ Intestinal tract is known to localize a large number of bacteria, with greater than 98% belonging to the phyla Firmicutes, Bacteroidetes, Proteobacteria and Actinobacteria, among which Firmicutes and Bacteroidetes are dominant. Accumulating evidence have proved that, as the major secondary bile acid, DCA is solely formed via 7α-dehydroxylation by a group of colonic bacteria that restrict to the genus *Clostridium*, especially bacteria within *Clostridium* clusters XI and XIVa, which possess the 7α-dehydroxylating activity.^25^ Consistent with previous findings, here we observed that the percentage of *Clostridium* belonging to the Firmicutes phylum (Gram-positive bacterial) expanded dramatically from less than 10% in controls to more than 60% in mice on a HFD, accompanied by increased fecal DCA and colonic pro-inflammatory macrophages infiltration, and our microbiome analysis noted that *Clostridium* clusters XI and XVIa increased obviously in HFD compared to normal-chow diet, thus might be responsible for the elevation of fecal DCA. Moreover, vancomycin (VCM) administration that targets Gram-positive bacteria dramatically decreased the fecal DCA level and ameliorated colonic pro-inflammatory macrophages infiltration in HFD mouse model, confirming that the Gram-positive bacteria related factors contribute to HFD-induced intestinal inflammation. Kawano et al. has revealed that it is the colon, but not the small intestine, firstly suffers from inflammation in response to HFD,^9^ based on this report and the fact that DCA is produced in colon and its concentration increases dramatically under HFD conditions, together with our *in vivo* findings showing that feeding the VCM-treated mice with HFD supplemented with DCA alone could significantly induce the colonic pro-inflammatory macrophages infiltration and reverse the beneficial effect of VCM treatment, these results highly suggest that microbial metabolite DCA could be an critical initiator for HFD-related colonic inflammation. Importantly, HFD-related high level DCA also contributes to the increased susceptibility to other factors induced intestinal inflammation according to our HFD-fed DSS-treated colitis model.

The DCA concentration used in our *in vivo* study was determined according to the previous report.^7^ To mimic the consequence of high fat diet, Bernstein and colleagues fed the mice a diet supplemented with 0.2% DCA to establish a colitis mouse model, which shares great similarities to human IBD. In our study, we observed that DCA-supplemented diet to VCM-treatment mice caused approximate twofold increase of fecal DCA compared to normal diet group (data not shown), and this DCA level is close to those following HFD treatments.

Lamina propria (LP) macrophages serve as the major contributors to the intestinal immune balance and they can respond to environmental cues promptly. It is reported that macrophage activation and infiltration play an central role in HFD-induced inflammation in the colon.^9,17^ Here we proved that DCA promoted M1 polarization of macrophages and release of pro-inflammatory cytokines, such as IL-6 and TNF-α, which could further aggravate macrophages activation and infiltration. In addition, HFD has been reported to increase the colonic expression of Ccl2, which was proved to be a key molecule mediating pro-inflammatory macrophages infiltration in colon. Consistently, our *in vitro* study found that DCA could significantly up-regulate Ccl2 expression in both macrophage and colonic cell line in a dose-dependent manner (data not shown), which implies the important role of excessive DCA in the recruitment of colonic macrophages under the HFD settings, together with our peritoneal injection data exhibiting that DCA administration elicited a considerable increase in the recruitment of macrophages, thus these findings indicate that DCA crucially contributes to the HFD-induced pro-inflammatory macrophages infiltration and colonic inflammation.

As signaling molecules, bile acids functionally exert diverse effects via corresponding receptors. Here our data showed that the major bile acid receptors, known as TGR5 and FXR, are not involved in the induction of M1 macrophage polarization by DCA. Instead, M2 muscarinic acetylcholine receptor (M2-mAchR), which has been reported to be the receptor of conjugated bile acid, participates in the DCA induced macrophage activation. Previous studies have shown that taurocholic acid could induce fetal arrhythmia associated with the cholestasis of pregnancy by signaling through M2-mAchR, thereby establishing the linkage between bile acids and acetylcholine receptor.^37^ Our novel observations revealed that free bile acid DCA could interact with M2-mAchR and then transactivate TLR2, a major pattern recognition receptor (PRR) that recognizes the conserved pathogen-associated molecular patterns (PAMPs), therefore triggers the downstream MAPKs and NF-kB signaling cascade to elicit the inflammatory response. TLR2 has been known to play a key role in the recognition of components of Gram-positive bacteria such as peptidoglycans,^38^ here our results disclosed a new scenario that DCA, a Gram-positive bacteria metabolite, could also functions as a PAMP/DAMP and be perceived by TLR2. Thus, innate immune receptor TLR2 can be responsive to diverse dangerous alteration of gut microenvironment. Intriguingly, DCA induces prompt TLR2 activation by upregulating TLR2 phosphorylation, meanwhile, increases TLR2 expression level via targeting AP-1 transcription factor and thus may guarantee the sustained TLR2 signaling transduction. TLR2^+^ macrophages population was previously found to predominate in the inflamed colon and could produce inflammatory mediators, whereas TLR2 ^–^ macrophages mainly resided in resting colon and unable to produce inflammatory mediators,^39^ this fact, together with our findings supports the contribution of high level DCA to the initiation of colonic inflammation. In addition, M2-mAchR/Src signaling was found to be involved in DCA induced TLR2 tyrosine phosphorylation, an important parallel phenomena was described for EGFR, which is also transactivated by bile acids in a muscarinic receptor-dependent or TGR5-mediated manner and contributes to the cancer cell proliferation and survival,^24,30,40^ thus crosstalk between bile acid receptor and other co-expressed receptors may represent an important fashion that bile acids play their diverse roles, and the regulation of intermediate process should be further elucidated.

Additionally, so far as inflammation is concerned, previously we found that DCA could activate NLRP3 inflammasome via S1PR2/Cathepsin B pathway in macrophages.^41^ In the current study M2-mAchR was observed to be involved in the polarization of M1 macrophage and production of pro-inflammatory factors, including TNFα, IL-6 and iNOS, induced by DCA largely through TLR2 signaling. Based on the previous research, M1 macrophages polarization is commonly accompanied by inflammasome activation, for instance, HMGB1 was reported to participate in the pathogenesis of acute lung injury by inducing M1 macrophages polarization and activating AIM2 inflammasome.^42^ IFN-γ treatment, leading to M1 phenotype of macrophages, could subsequently induce mRNA expression and active protein formation of caspase-1, a critical inflammasome component.^43^ In addition, NLRP3 inflammasome activation has been suggested to be initiated by Toll-like receptors (TLRs). Since NLRP3 inflammasome complex assembles upon receiving two signals, a priming signal commonly from TLRs-driven NF-kB activation and an activating signal due to the potassium efflux or ATP release, DCA-triggered TLR2 activation via M2-mAchR may provide priming signal for NLRP3 inflammasome activation, while generated IL-1β could serve as a stimulus for further macrophage polarization. Although the interaction between S1PR2/Cathepsin B and M2-mAchR/TLR2 pathways is not clearly understood, considering the close relationship between pro-inflammatory cytokines (TNF-α, IL-6 and IL-1β) and the occurrence of colitis, these pathways may both contribute to the high level DCA induced inflammatory injury in colon.

Many findings have demonstrated a direct effect of DCA on intestinal epithelial cells. Indeed, high level DCA (800 μM or greater) has been shown to decrease the transepithelial electrical resistance (TER) of enterocyte monolayers, indicating the intestinal epithelial barrier dysfunction.^44^ Even higher concentration of DCA (at millimolar levels) was required in the *in vivo* studies to exhibit dose-related increase of paracellular mucosal permeability in the intestine.^45–47^ Under high fat diet conditions, fecal DCA level was reported to be about several hundred micromols (commonly 200 ~ 300 μM) that obvious epithelial barrier damage could not be caused. The concentration of DCA in the colonic mucosal lamina propria remains ill defined, since one third to a half of colonic DCA undergoes re-absorption, the estimated colonic lamina propria DCA may reach to ~100 μM or more accordingly which could activate macrophages. It cannot be excluded that repeated long-term exposure of colonic epithelial cells to relatively high levels of DCA could induce epithelial barrier injury, although the activation of macrophage by DCA is suggested to contribute to the colonic inflammation at early initiating phase under HFD condition. Therefore, the combined effect of DCA on macrophages and epithelial cells may coordinately deteriorate colonic injury from a long perspective.

In summary, our findings reveal that excessive microbial metabolite DCA accumulating in the colonic lamina propria constructs a pro-inflammatory biased intestinal microenvironment under HFD conditions which could be sensed by macrophages through M2-mAchR/TLR2 and drive inflammatory response, thus highlight the critical role of microbial bile acid metabolite on innate immune system activation in the initiation of HFD-induced colonic and subsequent systemic inflammation. Moreover, HFD-related macrophage polarization and meta-inflammation are further involved in the pathogenesis of multiple inflammatory disorders, such as insulin resistance and atherosclerosis. Therefore, our data suggest a mechanistic basis that limiting the effect of DCA by modulation of gut microbiota or using BA sequestrants as well as targeting specific bile acid receptor signalings in macrophages may represent promising preventive and therapeutic measurements for colitis and other HFD-related inflammatory diseases.

## Materials and methods

### Animal treatment

C57BL/6 male mice (6- to 8-week-old) were purchased from Experimental Animal Center of the Chinese Academy of Sciences (Shanghai, China) and maintained in a specific pathogen free facility. All the animal experiment procedures complied with the Guide for the Care and Use of Medical Laboratory Animals issued by the Ministry of Health of China and approved by the Shanghai Laboratory Animal Care and Use Committee.

The mice were fed with normal diet (12kcal% fat) or high-fat diet (60kcal% fat) ad libitum for 20 weeks. For some HFD-fed mice, antibiotic treatment targeting Gram-positive bacteria was performed using vancomycin (500 mg/L) in drinking water from 9th week until sacrificed. Body weight was monitored weekly throughout the course of experiment. Fresh fecal samples were collected for the gut microbiome analysis through 16S rDNA amplicon sequencing or for the fecal DCA detection by LC-MS/MS analysis. Mice were sacrificed after 20 weeks feeding and colon tissues were harvested. The paraffin sections of murine colon were used for HE staining as well as immunohistochemical and immunofluorescent analysis. For the observation of DCA effect, vancomycin-administrated mice as described above were fed with HFD supplemented with 0.2% DCA from 9th week till the end of 20th week, and vancomycin-administrated mice with HFD served as control (n = 6/group).

### Histological analysis

Colonic histological scoring was determined as described previously,^48^ mainly by crypt architecture (0–5), crypt length (0–4), crypt abscesses (0–3), tissue damage (0–3), goblet cell loss (0–3), inflammatory cell infiltration (0–3) and lamina propria neutrophils (PMN) per high power field (scored from 10 fields) (0–3) in a blinded manner. The combined histological score ranged from 0 to 24.

### Reagents

Deoxycholic acid (DCA) and JTE-013 were purchased from Sigma-Aldrich (St. Louis, MO). Z-Guggulsterone was purchased from Santa cruz Biotechnology (Santa cruz, CA). Phospherylated and total ERK, JNK, IκB antibodies and SP600125, SB 203580, U0126 were purchased from Cell Signaling Technologies (Beverly, MA). Vancomycin, QNZ and SKI-606 were obtained from Selleckchem (Houston, TX). Methoctramine was purchased from AdooQ Bioscience (Irvine, CA). SR 11302 was purchased from APExBIO (Houston, TX). C29 was obtained from MedChemExpress (Monmouth Junction, NJ). TLR2, Src antibodies were from Abcam and phosphotyrosine antibody (4G10) was from Millipore. ELISA Kits were obtained from eBioscience (San Diego, CA).

### Cells

The murine macrophage cell line RAW264.7 was obtained from the Type Culture Collection of the Chinese Academy of Sciences (Shanghai, China). Cells were cultured in DMEM culture medium (Invitrogen) supplemented with 10% fetal bovine serum (Gibico) and 1% antibiotics (Invitrogen) at 37°C with 5% CO_2._ Bone marrow derived macrophages (BMDMs) were harvested from the femurs of C57BL/6 mice, and cultured in DMEM supplemented with 10% FBS and 50 ng/ml M-CSF (R&D Systems) for 6 to 7 days. Adherent cells with a purity of > 95% were used for the following experiments.

### Real-time PCR

Total RNA was extracted using a Pure Yield RNA Midi-prep kit (Promega) and then was reverse transcribed into cDNA with a Transcriptor First Strand cDNA Synthesis Kit (Roche). The expression of M2 muscarinic acetylcholine receptor was determined by regular PCR and the mRNA levels of iNOS, TNF-α, IL-6, CD206, arginase and TLR2 were evaluated by Real-time PCR on the PikoReal Real-Time PCR System (Thermo, Waltham, MA).

### NO measurement

Macrophages were stimulated by DCA for 24 h, then culture supernatants were collected and NO levels were measured using Griess reagents as previously described.^49^ Briefly, culture supernatants were mixed with an equal volume of Griess reagent (0.1% N-1-naphthylethylenediamine dihydrochloride and 1% sulfanilamide in 5% phosphoric acid) and incubated for 10 min at room temperature, then the absorbance was measured at 550 nm. Nitrite concentrations were determined according to a standard curve derived from the reaction of NaNO_2_ in the assay. For some experiments, various receptor or signaling pathway inhibitors (e.g. z-guggulsterone, JTE-013, methoctramine, C29, U0126, SP600125, SB203580) were added 30 min prior to DCA treatment.

### Peritoneal DCA injection

C57BL/6 mice (12 weeks) were intra-peritoneally injected with DCA (50 mg/kg) or vehicle. After 6 h, mice were sacrificed and peritoneal cavities were washed with 2 ml of PBS. The lavage cells were collected and analyzed for macrophage recruitment and polarization by FACS. Peritoneal macrophages were also isolated and cultured for 24 h, then the supernatants were harvested for TNF-α and NO detection.

### Flow cytometry

Peritoneal macrophages were incubated with an Fc blocking antibody (Clone 2.4G2, BD Biosciences) for 20 min at 4°C and then stained with anti-CD11b-PE, anti-F4/80-FITC antibodies for 30 min at 4°C. Intracellular staining was performed, if necessary, with anti-CD206-APC or anti-iNOS-APC antibody. Data were acquired on a FACSCanto II flow cytometer (BD Biosciences, San Jose, CA, USA) and analyzed by using FlowJo (Tree Star, Inc., Ashland, OR, USA).

### Transfection of small interfering RNA oligonucleotides

Macrophages were transfected with TGR5 small interfering RNA or scrambled siRNA in 6-well plates by using TransIT-Jurkat (Mirus Bio, Madison, WI), followed by DCA treatment (100 μM). TNF-α and NO levels in supernatant were measured accordingly. RNA oligonucleotides sequences were as follows: TGR5, forward 5ʹ-CUG GAA CUC UGU UAU CGC UTT-3ʹ and reverse 5ʹ-AGC GAU AAC AGA GUU CCA GTT-3ʹ. FXR, forward 5′-CCA AGA ACG CCG UGU ACA ATT-3′, reverse 5′-UUG UAC ACG GCG UUC UUG GTT-3′. S1PR2, forward 5′-CCU CUA CAA AGC CCA CUA UTT-3′, reverse 5′-AUA GUG GGC UUU GUA GAG GTT-3′. M2-mAchR, forward 5′-CCU CUA ACC UAC CCA GUU ATT-3′, reverse 5′-UAA CUG GGU AGG UUA GAG GTT-3′. TLR2, forward 5′-CCA AUC UCA CAA AUU UAC ATT-3′, reverse 5′-UGU AAA UUU GUG AGA UUG GTT-3′.

### Proteomic analysis

Bone marrow derived macrophages (BMDMs) were untreated or treated with DCA for 24 hours, then whole cellular lysates (WCE) were extracted using cell lysis buffer containing protease inhibitor for 30 min at 4°C and boiled at 100°C for 10 min in loading buffer. 80 μg of proteins were loaded on SDS-PAGE gel for separation. The gels were stained with Coomassie Blue R-250 to visualize the protein bands and each lane was cut into 5 gel slices, then in-gel trypsin digestion was performed at 37°C overnight. The digested products of each lane were extracted with acetonitrile (ACN) and dried in vacuum. Tryptic peptides were separated on a C18 column with a acetonitrile gradient (6, 9, 12, 15, 18, 21, 25, 30 and 35%). Dried peptide samples were re-dissolved in solvent A (0.1% formic acid in water) and Liquid chromatography – tandem mass spectrometry (LC-MS/MS) analysis was performed with Easy-nLC 1000-Q-Exactive HF system (Thermo Fisher Scientific). Thermo raw data were processed using Mascot software (version 2.3.2). Protein identification and quantification were performed as described elsewhere.^50^ All the raw data can be accessed from iProX (http://www.iprox.org) with the identifier IPX0000377000. Protein intensity normalization, missing value imputation and differential analysis were determined using Prostar version 1.14. Statistical significance was determined by welch’s t-test, and *p* < .05 was considered statistically significant. KEGG analysis was performed using DAVID tools.

### Western blot

Macrophages were incubated with or without DCA for indicated time point, and the cells were lysed in a lysis buffer supplemented with protease and phosphatase inhibitors (Theromo Fisher Scientific), then cell lysates were resolved by SDS-PAGE, transferred to 0.2 μm PVDF membranes and probed with antibodies against phospho-ERK (*p*-ERK), *p*-IκB, *p*-JNK (Cell Signaling) or *p*-Tyr416-Src, TLR2 (Abcam). Membranes were then incubated with a stripping buffer (Thermo Fisher Scientific) for 30 minu and re-probed with antibodies against total ERK, JNK, IκB (Cell Signaling), Src (Abcam), and β-actin (sigma). Reactive signals were detected by enhanced chemiluminescence (Thermo Fisher Scientific) and quantified by ChemiDoc™ XRS^+^ System (Bio-Rad).

### Luciferase assay

Murine TLR2 promoter region (2000bp) was amplified by polymerase chain reaction (PCR) and cloned into the Firefly luciferase reporter plasmid pGL3-Basic (Promega) using EcoR I and Hind III restriction sites. RAW 264.7 cells seeded on 48-well plates were incubated until 60% to 70% confluence and then were transient transfected with 0.4 μg pGL3-TLR2 plasmid using TransIT – Jurkat Transfection Reagent (Mirus Bio). After transfection overnight, cells were left untreated or treated with DCA (100 μM) for 24 h, and methoctramine (5 μM) or SR 11302 (100 μM) was added 30 min ahead of DCA treatment if necessary. Cell lysates were collected for Luciferase activity determination by Dual Luciferase Reporter assay systems (Promega). According to the manufacturer’s instructions, luciferase activity was normalized by Renilla activity to standardize transfection efficiency. For the deletion analysis, a series of deletion mutants of the TLR2 promoter reporter plasmids (−1500, −1025, and −524/-1 bp) were constructed and applied.

### Chromatin immunoprecipitation assay

The ChIP procedure was performed with an assay kit (Beyotime) according to the manufacturer’s protocols. After treatment as indicated, RAW264.7 cells were cross-linked by 1% formaldehyde, then nuclei were harvested and subjected to sonication. Chromatin extracts were pre-cleared with protein A + G agarose and immunoprecipitated with c-Jun antibody (Cell Signaling Technology) or rabbit IgG (negative control) overnight at 4°C. De-crosslinking was then conducted followed by DNA purification. Finally, the purified DNA underwent PCR amplification using primers (forward: 5ʹ – TGA CAA CCT ATA AGG ACA AGG GAT G-3ʹ, and reverse: 5ʹ – CAA GGC GAG ATT GGA TGC AGA GCA A-3ʹ) encompassing the AP-1 binding region of the murine TLR2 promoter.

### Immunoprecipitation

Macrophages were stimulated with DCA (100 μM) for indicated time point and whole cell lysates were prepared using the Mammalian Cell Lysis kit (Sigma-Aldrich). 200 μg of protein was incubated with TLR2 antibody (Abcam) for 1 h at 4°C followed by incubation with protein A/G beads (40 μl) overnight at 4°C. Beads were then washed five times with lysis buffer and the supernatant was carefully removed. 50 μl of 1 × western blot loading buffer was added to the bead pellet and heated at 90–100°C for 5 min. The mixture was centrifuged at 10,000 g for 5 min and the supernatant was collected and subjected to SDS-PAGE. Protein was then transferred to the PVDF membrane and the immunobloting was performed using 4G10 antibody (Millipore).

### Statistics

Results were expressed as mean ± standard error of the mean (s.e.m.). Data was compared by using 2-tailed Student’s *t*-test or one-way analysis of variance (ANOVA). *p* < .05 were considered to be significantly different.

## Supplementary Material

Supplemental Material
