# Supplementary material for: Gut microbial bile acid metabolite skews macrophage polarization and contributes to high-fat diet-induced colonic inflammation
Source: Gut Microbes. 2020 Oct 2;12(1):1819155. doi: 10.1080/19490976.2020.1819155 (PMC7553752; doi:10.1080/19490976.2020.1819155)
Supplement: Supplemental Material [file KGMI_A_1819155_SM6612.doc]

**Supplementary Data**

**Gut Microbial Bile Acid Metabolite Skews Macrophage polarization and Contributes to High-Fat Diet-Induced Colonic Inflammation**

**Lingyu Wang, Zizhen Gong, Xiuyuan Zhang, Fangxinxing Zhu, Yuchen Liu, Chaozhi Jin,** **Xixi Du,** **Congfeng Xu,** **Yingwei Chen,** **Wei Cai, Chunyan Tian, Jin Wu**

^*^**Address****Correspondence to**: Jin Wu, Phone: +86-21-25076443, Fax: +86-21-65791316, E-mail: wujin@xinhuamed.com.cn; Chunyan Tian, E-mail: tianchunyan@mail.ncpsb.org or Wei Cai, E-mail: caiw204@sjtu.edu.cn

**Inventory of Supplementary Data**

Supplementary Figures and Legends

- - - Supplementary Figure 1. HFD-related high level intestinal DCA contributes to the increased susceptibility of mice to DSS-induced colonic injury, related to Figure 1 and 2
    - Supplementary Figure 2. DCA administration promotes peritoneal macrophage recruitment and M1 polarization, related to Figure 3
    - Supplementary Figure 3. The effect of DCA and M2-mAchR/AP-1 inhibitors on macrophages viability, related to Figure 3 and 6
    - Supplementary Figure 4. M2-mAchR and TLR2 are involved in DCA-induced macrophage polarization, related to Figure 4 and 5
    - Supplementary Figure 5. Relative quantification of ChIP assay, related to Figure 6f.

**Supplementary Figures and Legends**

**
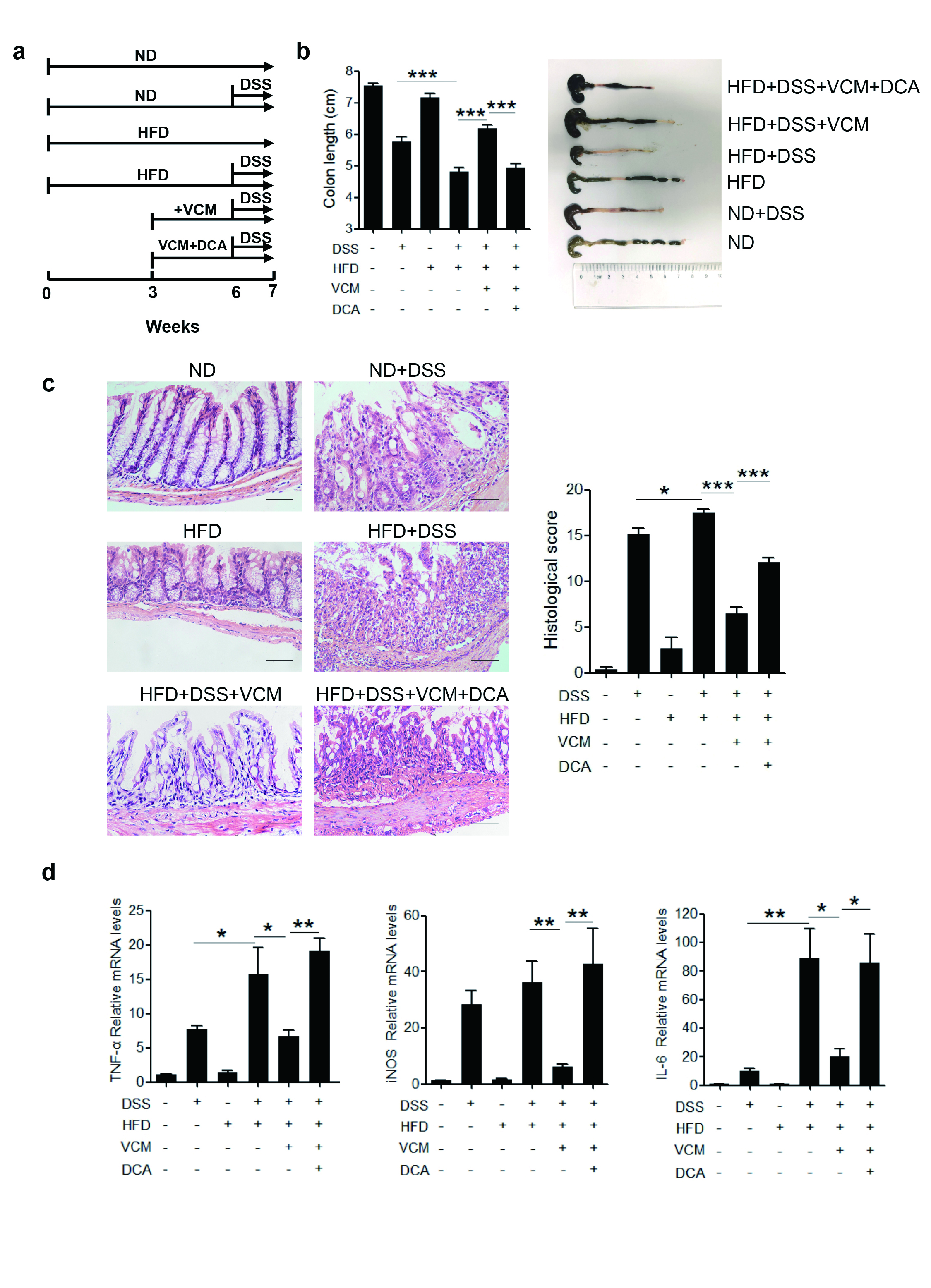
**

**Supplementary Fig. 1** HFD-related high level intestinal DCA contributes to the increased susceptibility of mice to DSS-induced colonic injury. (a) Animal treatment procedure (n=6 per group). (b) Colon length. (c) Representative HE staining and histological score of colon sections of differently treated mice (Scale bar, 50µM). (d) Real-time PCR analysis of TNF-α, iNOS and IL-6 levels in colonic homogenates of differently treated mice. *: p < 0.05; **: p < 0.01; ***: p < 0.001. Error bars indicate s.e.m.

**
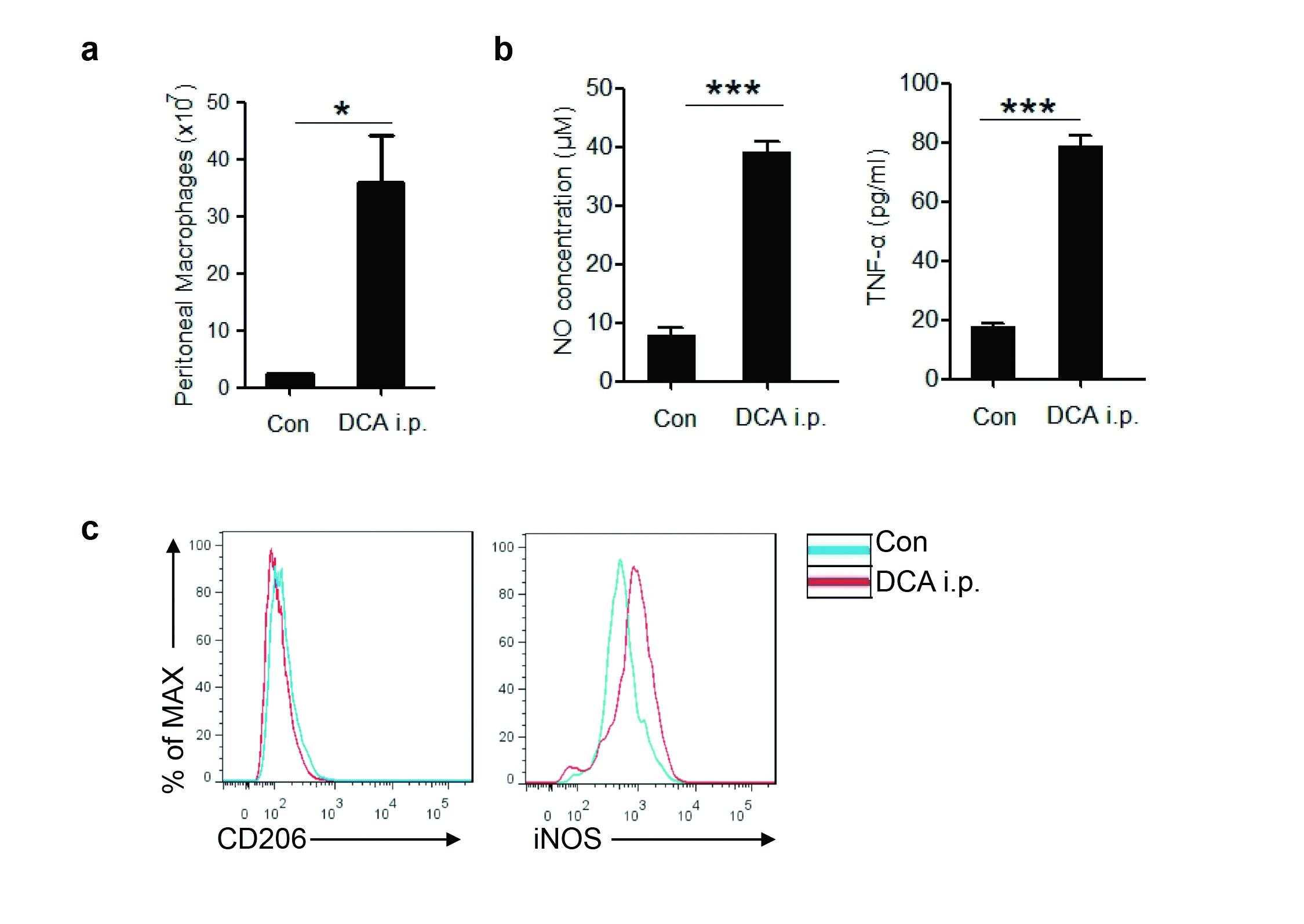
**

**Supplementary Fig. 2**  DCA administration promotes peritoneal macrophage recruitment and M1 polarization. Mice were intra-peritoneally injected with DCA or vehicle, **a** Statistical comparison of peritoneal macrophage numbers in vehicle- or DCA-injected mice. **b** NO and TNF-α production by peritoneal macrophages in vehicle- or DCA-treated mice. **c** Flow cytometry analysis of CD206 and iNOS expression by peritoneal macrophages in vehicle- or DCA-treated mice. *: p < 0.05; ***: *p* < 0.001.


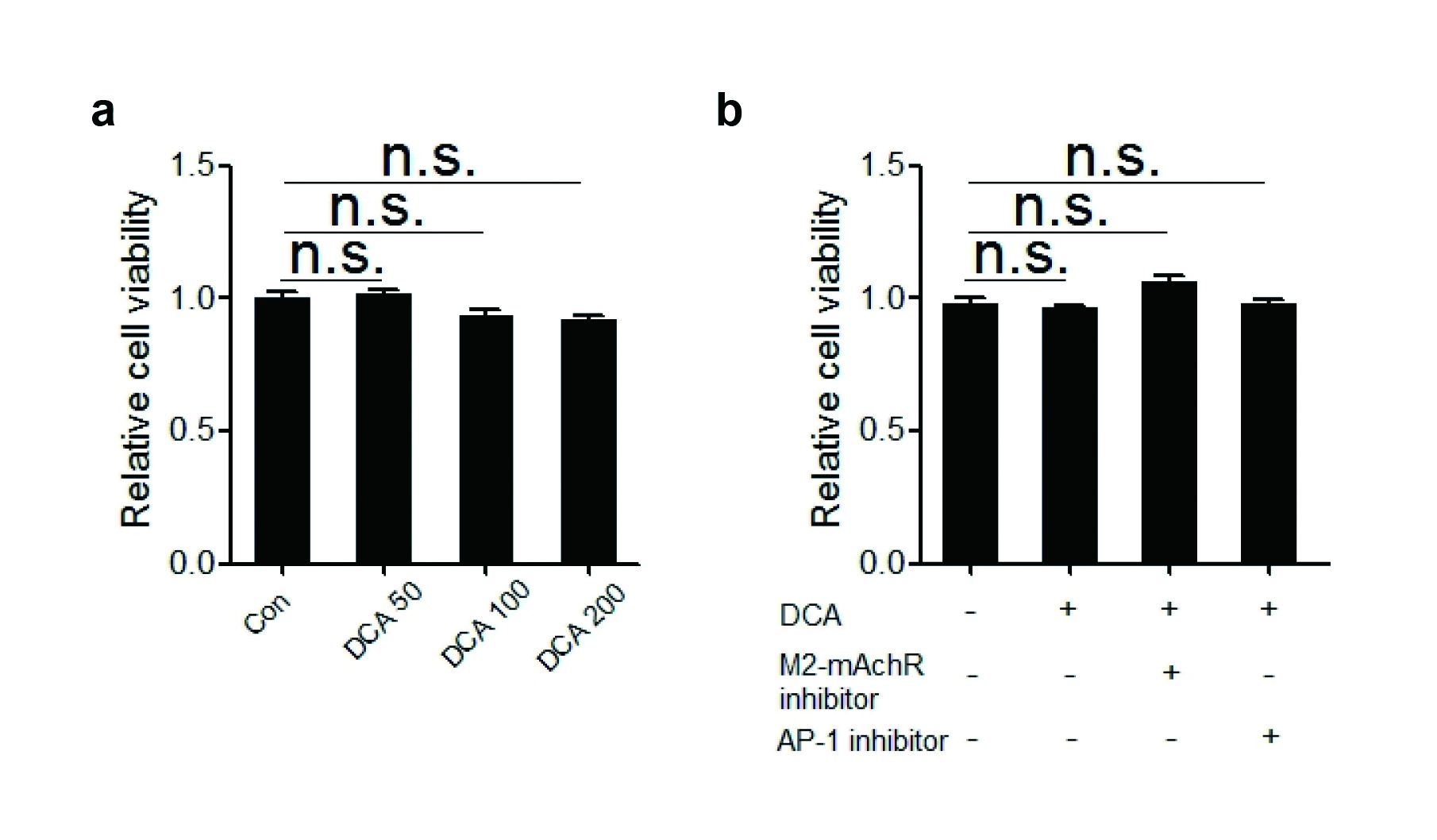


**Supplementary Fig. 3** The effect of DCA and M2-mAchR/AP-1 inhibitors on macrophages viability. (a) Relative cell viability of macrophages treated with different dosages of DCA was analyzed by CCK-8 assay. (b) Relative cell viability of macrophages treated with DCA (100μM) in the combination of M2-mAchR or AP-1 inhibitors. n.s.: no statistically significant difference (p > 0.05). Data from 3 independent experiments are shown. Error bars indicate s.e.m.


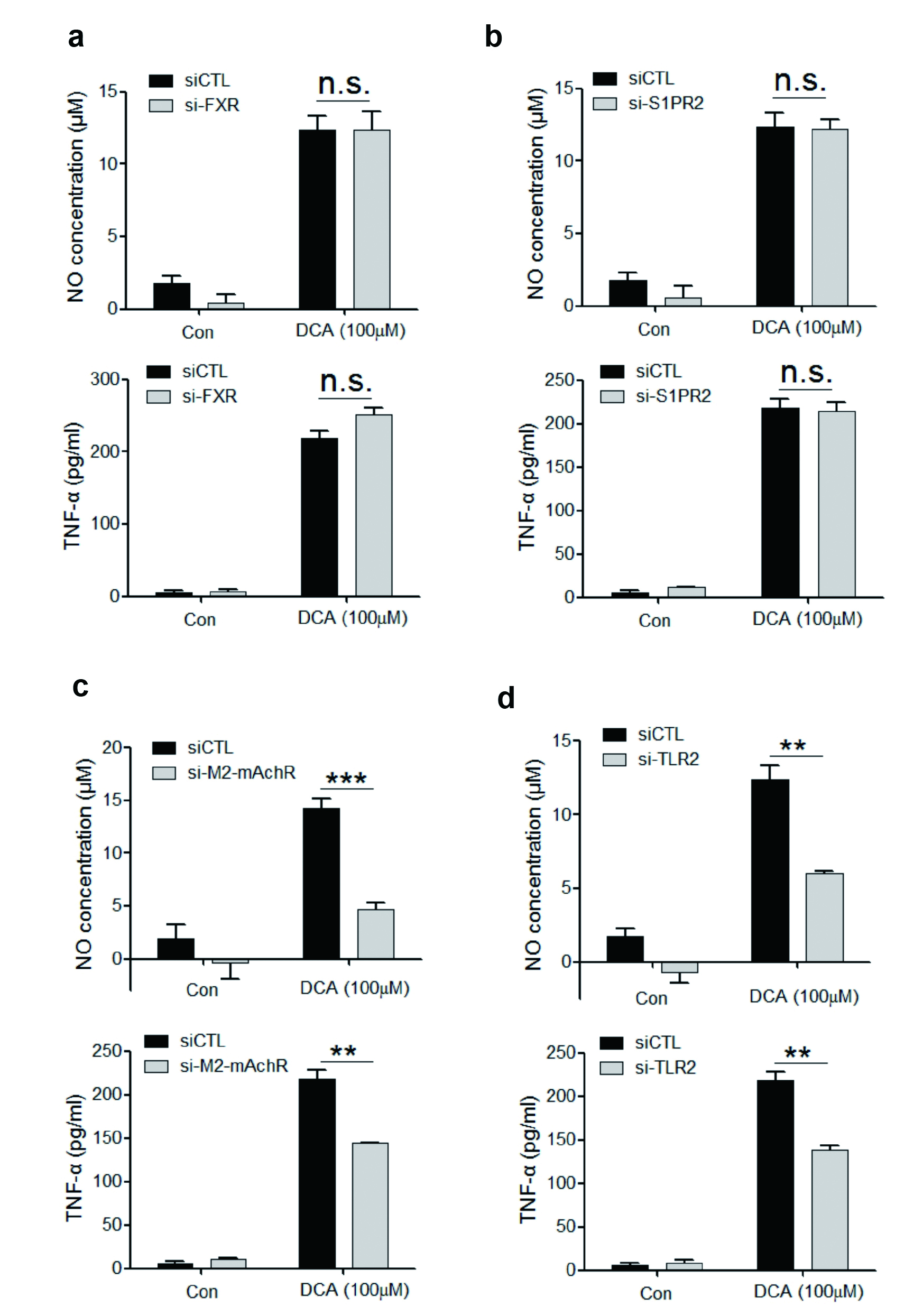


**Supplementary Fig. 4** M2-mAchR and TLR2 are involved in DCA-induced macrophage polarization. (a) Scramble control siRNA (siCTL) or FXR siRNA (si-FXR), (b) Scramble control siRNA (siCTL) or S1PR2 siRNA (si-S1PR2), (c) Scramble control siRNA (siCTL) or M2-mAchR siRNA (si-M2-mAchR), (d) Scramble control siRNA (siCTL) or TLR2 siRNA (si-TLR2) transfected RAW264.7 macrophages were stimulated with DCA (100µM). NO and TNF-α in supernatants were analyzed. **: *p* < 0.01; ***: *p* < 0.001. n.s.: no statistically significant difference (*p* > 0.05). Data from 3 independent experiments are shown. Error bars indicate s.e.m.


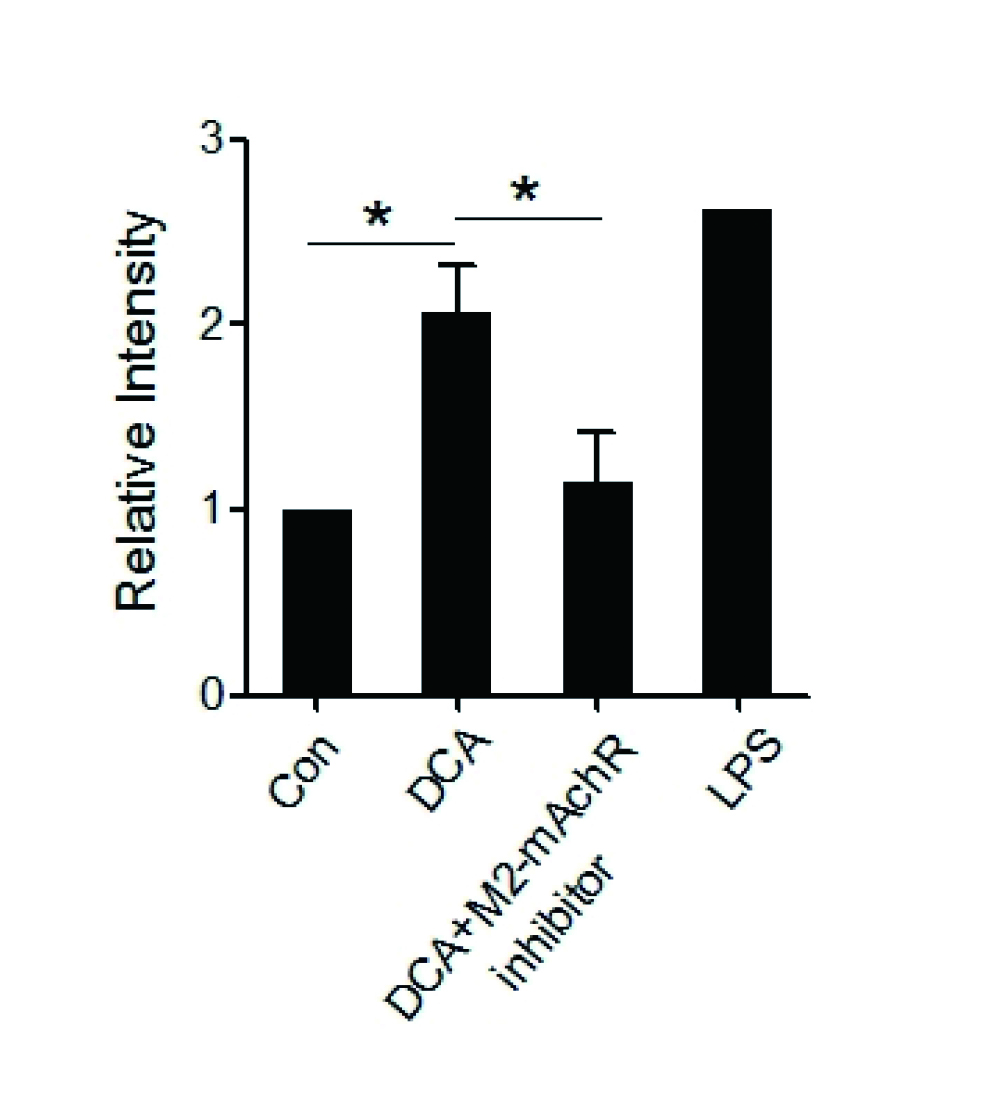


**Supplementary Fig. 5**  Relative quantification of ChIP assay in Figure 6f.
